# Comprehensive insights into a decade-long journey: The evolution, impact, and human factors of an asynchronous telemedicine program for diabetic retinopathy screening in Pennsylvania, United States

**DOI:** 10.1371/journal.pone.0305586

**Published:** 2024-07-12

**Authors:** Francisco J. Bonilla-Escobar, Anthony I. Ghobrial, Denise S. Gallagher, Andrew Eller, Evan L. Waxman

**Affiliations:** 1 Department of Ophthalmology, UPMC Vision Institute, University of Pittsburgh, Pittsburgh, Pennsylvania, United States of America; 2 Grupo de Investigación Visión y Salud Ocular, Servicio de Oftalmología, Universidad del Valle, Cali, Colombia; 3 Fundación Somos Ciencia al Servicio de la Comunidad, Fundación SCISCO / Science to Serve the Community Foundation, SCISCO Foundation, Cali, Colombia; University of Warmia, POLAND

## Abstract

Diabetic Retinopathy stands as a leading cause of irreversible blindness, necessitating frequent examinations, especially in the early stages where effective treatments are available. However, current examination rates vary widely, ranging from 25–60%. This study scrutinizes the Point-of-Care Diabetic Retinopathy Examination Program at the University of Pittsburgh/UPMC, delving into its composition, evolution, challenges, solutions, and improvement opportunities. Employing a narrative approach, insights are gathered from key stakeholders, including ophthalmologists and staff from primary care clinics. A quantitative analysis from 2008 to 2020 provides a comprehensive overview of program outcomes, covering 94 primary care offices with 51 retinal cameras. Program components feature automated non-mydriatic 45° retinal cameras, a dedicated coordinator, rigorous training, and standardized workflows. Over this period, the program conducted 21,960 exams in 16,458 unique individuals, revealing a diverse population with an average age of 58.5 and a balanced gender distribution. Average body mass index (33.96±8.02 kg/m2) and hemoglobin A1c (7.58%±1.88%) surpassed normal ranges, indicating prevalent risk factors for diabetes-related complications. Notably, 24.2% of patients underwent more than one exam, emphasizing program engagement. Findings indicated that 86.3% of exams were gradable, with 59.0% within normal limits, 12.1% showing some evidence of diabetic retinopathy, and 6.4% exhibiting vision-threatening diabetic retinopathy. Follow-up appointments with ophthalmologists were recommended in 31.5% of exams due to indeterminate results, positive diabetic retinopathy (≥moderate or macular exudate), or other findings like age-related macular degeneration or suspected glaucoma. The program demonstrated high reproducibility across diverse healthcare settings, featuring a sustainable model with minimal camera downtime, standardized workflows, and financial support from grants, health systems, and clinical revenues. Despite COVID-19 pandemic challenges, this research emphasizes the program’s reproducibility, user-friendly evolution, and promising outcomes. Beyond technical contributions, it highlights human factors influencing program success. Future research could explore adherence to follow-up ophthalmological recommendations and its associated factors.

## Introduction

Diabetic Retinopathy (DR) is a leading cause of irreversible vision loss [1]. While there are effective treatments for DR, these are most successful in the early stages when patients are asymptomatic [2–6]. Consequently, patients with diabetes should be examined annually for DR [7]; however, this currently occurs at rates between 25–60% [7–10].

Known barriers to examination include finances, transportation, lack of education, time constraints, and access [8, 11–13], all of which are more prevalent in people living in underserved communities in which diabetes, diabetic complications, and DR are more prevalent [14].

The standard for DR examination is an in-person retinal examination by an ophthalmologist or optometrist through dilated pupils [15, 16]. Retinal photography with remote interpretation has been used as a method of overcoming barriers to an in-person examination, demonstrating to be sensitive, specific, and cost-effective [9, 12].

There are several programs with remote interpretation of retinal photographs of diabetic patients in the US and abroad [8, 9, 17–31]. The American Teleophthalmology Association (ATA) has created technical guidelines for teleophthalmology programs, including those for DR [32]. Programs using remote interpretation for DR examination (DRE) are often described from a technological standpoint in terms of imaging technology, image transmission, and interpretation platform, and in term of outcomes emphasizing the quality of images and DR diagnosis and classification [18, 19, 21].

The implementation of an effective, scalable, and sustainable program for photographic retinal examination must consider the needs of patients, physicians, staff, ophthalmologists, and payors in the context of existing systems of documentation, communication, and referral patterns [32, 33]. We document the current composition, workflow, and outcomes (participants characteristics, quality of images and diagnosis) of the Point-of-Care Diabetic Retinopathy Examination Program (POCDREP) at the University of Pittsburgh/UPMC, and describe the challenges faced as the program grew, solutions to those challenges, and opportunities for continued improvement.

## Methods

In this study, we provide a narrative approach to describe the implementation of the POCDREP, its evolution, and its status. A quantitative approach is used to report program outcomes based on a retrospective chart review. The period reported on includes the exams carried out from September 1^st^, 2008, to December 31^st^, 2020.

### Description of the program and its evolution

As part of a quality improvement project (Code 630, July 5^th^, 2016), we interviewed key stakeholders—specifically, two ophthalmologists involved in program development, primary care clinic managers, and 31 primary care physicians—to identify barriers and facilitators to program implementation. Participants were selected based on their history of involvement in the program’s implementation within their respective clinics. Surveys were conducted from July 5^th^, 2016, and June 30^th^, 2017. Interview participation was voluntary, with verbal informed consent obtained.

**Fig 1:**
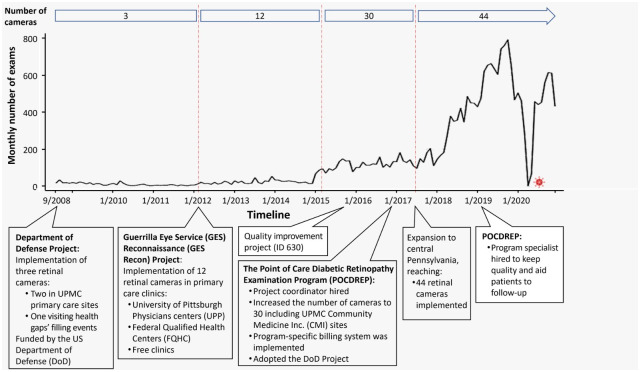
Timeline of the program. A narrative was formulated to depict pivotal moments and the rationale for changes in the program. This narrative is tailored for readers interested in implementing or enhancing a comparable program within their organization.

We also reviewed communications, reports, guides, and instruction materials to present a description of the program’s evolution, coupled with the construction and annotation of a timeline ranging from program predecessors and early efforts to the current state of the program (Fig 1).

### Program outcomes

Following the approval by the Institutional Review Board of the University of Pittsburgh on May 22^nd^, 2020, under the code STUDY20010159 (S1 File), we conducted secondary data collection. This process involved gathering data from medical records and the program’s automatic monthly reports from June 1^st^, 2020, to May 1^st^, 2021. Our focus was on exam results and basic sociodemographic and clinical information. Data from exams carried out between September 1^st^, 2008, and June 14^th^, 2015, from both UPMC and participating non-UPMC sites, were manually collected from their respective EHR systems. Data from June 15^th^, 2015, to December 31^st^, 2020, were gathered from automatic monthly reports generated by the UPMC Electronic Health Record (EHR) system (Epic). All data were consolidated into a unified database covering the study period.

### Variables

Analyzed data included retrospectively collected information about the (1) participating sites (location, number of offices using a camera, clinic affiliation to a healthcare system—i.e., UPMC, Federally Qualified Health Center (FQHC)—, clinic main specialty, and ordering provider), (2) patients´ characteristics (age, gender, ZIP code, most recent HgbA1C level, smoking status, race & ethnicity, and primary language spoken), and exam characteristics (date of the image, date of reading, image quality, findings and diagnosis, and recommendations). One participant could have more than one DRE during the analyzed period; therefore, exams and participants were analyzed separately.

We created a poverty level variable using information from the “Percentage of Individuals Living Below the Federal Poverty Level” based on the ZIP code from the Census 2000 Summary File 3 [34, 35]. Poverty status was categorized in quintiles in ascending order from the lowest to highest levels of poverty in the neighborhood as follows: <10% (Q1), 10–19.9% (Q2), 20–29.9% (Q3), 30–39.9% (Q4), >40% (Q5). Cut points were based on the US Census Bureau’s definitions of poverty areas [36].

Image graders assessed and categorized fundoscopic image quality for each eye as “good,” “fair,” “poor,” or “non-gradable.” If an image was not uploaded, it was graded as “no image.” Photographs without smears, artifacts, or movement were defined as “good” quality images. A “fair” picture entailed a degree of smear or artifact, but was of sufficient quality to be graded confidently. Images were graded as having "poor" quality if artifacts would have prevented an assessment of the absence of retinopathy but nevertheless demonstrated pathology. In images that were “non-gradable,” graders could not evaluate whether the region of the eye that should be photographed was within normal limits.

DRE findings were described based on DR severity levels as “within normal limits”, “mild non-proliferative DR (NPDR),” “moderate NPDR,” “severe NPDR,” and “proliferative DR” [37]. Another relevant finding was the presence of “macular exudate,” defined as a surrogate marker of clinically significant diabetic macular edema (DME) [38, 39]. Exams identified with DR were further classified as with vision-threatening DR (VTDR: macular exudate or ≥ moderate non-proliferative DR) or without VTDR. Graders also reported incidental findings and diagnoses, including suspected glaucoma, age-related macular degeneration (AMD), hypertensive retinopathy, among others [40].

Exams were classified as positive, indeterminate, with other findings requiring follow-up, or within normal limits. Positive exams were those with levels of DR severity ≥ moderate or with macular exudate. Exams with “non-gradable” image(s) or with “no image,” were classified as indeterminate for DR. Other findings such as suspected glaucoma, AMD, vein occlusions, nevus, and epiretinal membranes, among others, were classified as other findings requiring follow-up.

As the purpose of this study is to describe the program and its longitudinal outcomes, we used descriptive statistics to present outcomes based on data characteristics. Hence, we are describing the characteristics of (1) primary care clinics, (2) participants, and (3) exams.

We used frequencies and percentages for categorical variables and central tendency and dispersion measurements (mean and standard deviation, SD, or median and interquartile range, IQR) for quantitative variables. All analyzes were carried out in Stata16^®^ (StataCorp, TX).

## Results

### 1. Program structure

#### 1.1. Origins and evolution of the program

The first attempt to use remote photography to address diabetic eye examination care gaps at UPMC was in 2008 (Fig 1). Four retinal cameras, Topcon TRC NW400, were installed in various settings, including three in primary care offices and one in a mobile unit with funding from the US Department of Defense (DoD Project). However, the project faced limited success, despite efforts involving multiple focus group sessions with staff to facilitate the implementation of the cameras. However, participating sites engaged solely when compliance with the Healthcare Effectiveness Data and Information Set (HEDIS) measures was required.

The selected early non-mydriatic cameras, though operationally complex, were operated by non-ophthalmic technicians trained for image capture. These technicians also served as study coordinators in the feasibility research protocol, managing also consent forms. Challenges in camera maintenance resulted in suboptimal image quality, and the infrastructure for transmitting and reporting images was cumbersome, causing delays in interpretation. By 2012, only one primary care practice had a functioning camera, and the mobile unit was mainly used for DR exams at health fairs. The DoD Project concluded with approximately 750 patients screened for diabetic retinopathy.

In 2006, our department initiated a community outreach project to address unmet eye care needs in Pittsburgh and the surrounding areas. The Guerrilla Eye Service (GES) provides comprehensive eye examinations—refraction, anterior segment examination, dilated retina examination, and perimetry—to patients at free care clinics. GES is staffed by medical students, ophthalmology residents, and attendings. The service remains active in clinical sites around Pittsburgh. A large proportion of the patients served by GES have diabetes and in addition a there is a substantial no-show rate at the missions.

High no-show rates led to the idea that permanently stationing a non-mydriatic fundus camera at a GES site might improve exam rates and DR detection, as photographic exams would be captured when patients were seen for a medical exam. Automated, non-mydriatic cameras were becoming available, and with the help of a local foundation, a Centervue DRS^®^ nonmydriatic automatic camera was purchased for the McKeesport 9^th^ Street clinic in 2012. McKeesport’s population is a heterogeneous mix of ethnicities and nationalities, with 30.3% living below the poverty line [41].

In contrast with the earlier non-mydriatic retinal cameras and the high-end ophthalmic cameras, the Centervue DRS required minimal training for clinic staff. The transmission of images for reading and interpretation was conducted utilizing Centervue’s cloud-based reading platform under a subscription arrangement.

Over the following year, a neighboring primary care clinic periodically borrowed and used the camera; eventually, funding was secured to purchase two additional cameras in McKeesport, one of which was placed at the Latterman Family Health Center, with the other at the McKeesport Hospital Internal Medicine clinic. Both sites are UPMC facilities and share the UPMC EHR infrastructure.

The program’s success at indigent care sites in McKeesport was shared with the leadership of the Internal Medicine clinics at UPMC flagship sites in Oakland and Shadyside (UPMC University of Pittsburgh Physicians, UPP), where DR exam rates were approximately 20%. After a trial period with a loaned camera in 2014, UPMC purchased a camera for each flagship site.

The annual subscription to the cloud-based reading platform was deemed financially unsustainable. Because of this, the platform was eventually discontinued. The program moved to secure-email based interpretation or UPMC Epic EHR transmission of images and interpretations.

After the growth in McKeesport, the program received much greater interest, and group of three UPMC Family Medicine sites (St. Margaret Family Medicine) applied for a grant following a loaned trial, subsequently receiving funds for a camera at each site. Federal Qualified Health Centers (FQHC) that were part of the GES missions also acquired cameras.

In 2015, the remaining cameras from the DoD funded program were rolled into the point-of-care diabetic retinopathy examination program (POCDREP). Over this period, cameras were placed in additional non-UPMC indigent care sites around Pittsburgh [42], and a coordinator was hired to work on quality improvement for the program.

UPMC Community Medicine Inc. (UPMC CMI), the community-based medicine arm of UPMC, acquired 30 cameras for their sites. Over the next several years, the program reached sites outside the Pittsburgh region. Once again, having trialed the concept with a loaned camera, four clinics in Harrisburg, PA—about 200 miles from Pittsburgh—purchased cameras. In addition, the program now serves Erie, PA (~130 miles from Pittsburgh) and Susquehanna, PA (200 miles from Pittsburgh).

The program continues to grow, primarily by word of mouth between medical directors, who are then put in contact with our program, and loaned a camera for trial purposes. Following this, funding is obtained from UPMC or a local health-focused neighborhood foundation when success is demonstrated.

#### 1.2. Retinal cameras

Automated, non-mydriatic retinal cameras have been essential to the program’s success, primarily because they are easier to use, resulting in greater staff acceptance, albeit at the expense of the adjustability and versatility of the cameras intended for an ophthalmologist’s office. Clinic staff utilized either the Centervue DRS or Topcon TRC NW400 digital retinal camera to capture macula-centered, 45-degree images of each eye. Images from UPMC offices were stored and forwarded using Epic^®^, whereas those from non-UPMC facilities were forwarded via secure email. There are currently many cameras available, with each one requiring an investment of approximately $17,000. Currently, all but one of the cameras used in the UPMC program were manufactured by Centervue (now iCare). We have found that using a single camera model at nearly all sites simplifies deployment, configuration, training, and fosters peer support for troubleshooting. Cameras requiring repair can be replaced by a drop-in loaner camera from the program, with minimal downtime.

#### 1.3. Personnel

Initially, a department faculty member (EW) managed the program’s administration with support from department administrative assistants. However, as the program grew, it became evident that additional administrative support was required. As a result, a graduate student (FJBE) was recruited to serve as the program coordinator. Salary support for the coordinator position was secured from a grant from the Beckwith Institute, an endowed fund of UPMC, and subsequently from department funds as the program became large enough to generate sufficient revenue.

The coordinator is responsible for:

- Providing training to working personnel at new sites;
- Serving as a clinical site liaison to troubleshoot and train new personnel; and
- Working with local site personnel to arrange follow-up for patients with photographic exams that suggest the need for an in-person evaluation with an eye care provider.

At each clinical site, it has been essential to identify and engage an attending physician and office manager responsible for local oversight of the program. At each site, providers must understand the program, indications, and procedures for ordering photographic examinations; likewise, it is crucial that they familiarize nurses and medical assistants with the EHR workflow, and the camera’s operation.

#### 1.4. Training

Early in the program, we depended exclusively on representatives from the camera vendor to provide on-site training for the camera operation. While this was helpful, we found that it was insufficient as despite its relative ease of use, it became clear that it was necessary to provide multiple initial training sessions and further refresher training to help clinic staff routinely capture images of acceptable quality. In addition, training was required to implement the workflow specific to other EHRs. To ensure competence, the minimum requirements were one hour of online training, and a further hour in person; however, the trainer remained on site for a minimum of two days following this initial period to conduct further hands-on training, role play, and to provide in-person advice and recommendations.

Local clinic personnel were essential in the creation and iterative improvement of training materials that facilitated the acquisition of acceptable quality photographs while avoiding common imaging pitfalls. In addition, these staff were equally helpful in designing and improving EHR workflows and training materials for onboarding new personnel.

#### 1.5. Sustainability

Program expenses included those related to equipment, deployment, and personnel. Equipment expenses included the camera and table purchase cost for each site, along with associated infrastructure adjustments like electricity or internet port installations, stools, replacement of chair or stool casters with bell glides, lens cleaners, and occasional repair costs. Late in the program, service contracts were routinely purchased with each new camera. Deployment expenses could have included the costs of networking each new camera, but these were routinely absorbed by UPMC and, as such, were not visible to the program. Personnel expenses were limited to the salary of the project coordinator. The program director and clinic personnel did not receive additional compensation. Image graders were compensated as a result of additional generated clinical productivity.

Equipment costs were covered by grants or by the health system, and program revenues resulted from the clinical revenue attributed to clinical sites and image graders, clinical practice revenues, and healthcare payer revenues. The most significant, measurable revenue is from improved quality scores; additionally, the program may result in decreased costs to healthcare payers by detecting retinopathy early and preventing blindness. However, these cost savings are not easily calculated.

#### 1.6. Workflow

Epic is the EHR for outpatient care in the UPMC system. The workflow for DRE goes as follows:

- Patients are identified for inclusion in the program. On some sites, patients who meet the criteria for the program are identified on review of the daily patient schedule, and a photographic examination takes place as part of that visit. At other sites, the patient panel is reviewed regularly to determine which patients are overdue for DRE, following which they are called to schedule an exam specifically for this purpose.[15]
- An order for the photographic exam is placed in Epic;
- Clinic staff perform the photographic exam;
- Images resulting from the exam are uploaded to the Epic Media Manager and attached to the order;
- The order is forwarded to an Epic pool for interpretation of the photos;
- Two ophthalmologists in the reading pool, working from their Epic in-basket, examine each image and create a report;
- The report is entered into the interpretation section of the order and copied back to the ordering provider;
- The ordering provider is then responsible for following up with the patient and initiating any further actions as required.

**Fig 2:**
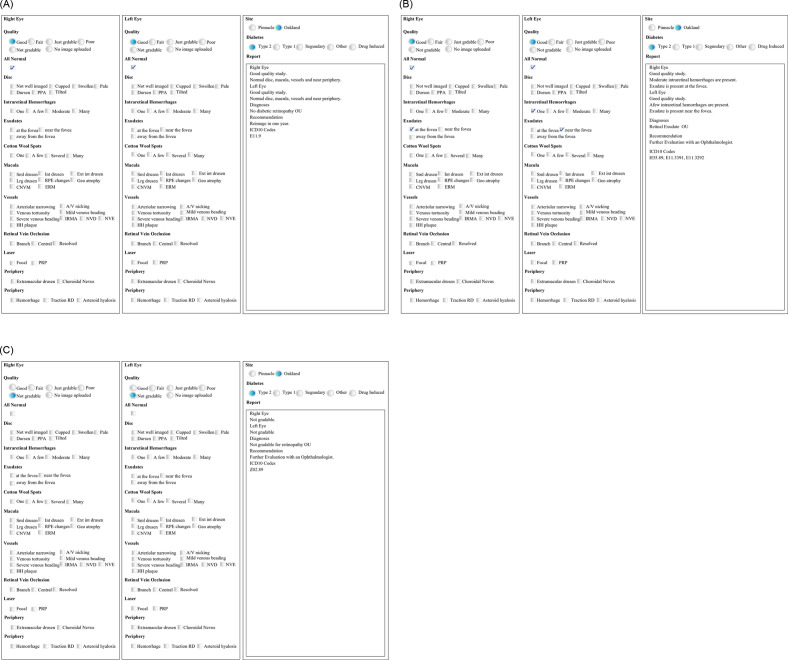
Program reading application and resulting system: (A) Within normal limits diabetic retinopathy exam, (B) Exam with abnormal results requiring a follow-up visit, (C) Non-gradable images.

Report creation is simplified and standardized with the help of software created in house. The physician using the software checks off findings in each eye, and the program then ‘calculates’ diagnoses and ICD-10 codes, determines a disposition, and creates a formatted report copied into Epic (Fig 2).

Photographic exams from sites that do not use Epic require additional steps at the beginning and end of the Epic workflow. Examination photos and patient data are communicated to the program coordinator via secure email and an Epic testing encounter is created to initiate the standard workflow. In addition to recording the interpretation in Epic, a secure email containing the report is sent to the provider.

#### 1.7. Coding and billing

A program-specific billing system was implemented in 2016. Decision-makers identified an opportunity for reimbursement after screening insured patients, with a reimbursement for both the clinic taking the photos and the graders. In addition, there are reimbursements and stimulus packages for sites improving their HEDIS measures, a widely used measure of care performance, and quality scores based on DRE rates. Once the program provided evidence of change in quality scores, the support from stakeholders increased. Creating a standard coding and billing infrastructure greatly facilitated the sustainability of program implementation.

#### 1.8. Patient follow-up

The need for documentation of patient follow-up after DRE was informed by the prevalence of patients who underwent multiple DREs without documentation follow-up. Within the first year of documentation (2016), we observed that the follow-up rate of patients who were recommended to seek further care with an eye doctor was 42%. This low rate suggested that DRE and diagnosis alone may not be sufficient to prevent DR progression, and that a more significant effort would have to be directed toward facilitating follow-up care after the initial DR diagnosis.

We helped enhance this process through a quality improvement project, instructing sites on correctly documenting the reports from community eye doctors in the EHR, asking primary care staff to address the reports, and noting instances of contact or attempts to contact the patient. Attempts to create an automated post-DRE tracking system in Epic were unsuccessful; consequently, post-DRE follow-up and tracking remain a manual process.

#### 1.9. COVID-19 pandemic

The program stopped imaging patients from March to May 2020 due to the COVID-19 pandemic and following this period, it was essential to identify how to safely restart the strategy. When using tabletop cameras, standard practice dictates cleaning the areas of patient contact before and after each patient, including the headrest, chinrest, and table. This practice did not change, with disposable alcohol swabs and wipes used to clean the surfaces. Upon site reopening, DRE numbers increased but have not yet returned to peak pre-pandemic levels.

Issues brought to our attention after the pandemic included the lack of in-person training, and staff being unable to recall specific configurations or procedures. Sites distant to Pittsburgh (+2 hours’ drive) were our main concern as the program was halted there for a longer period of time.

### 2. Program outcomes

#### 2.1. Participating sites

The program covers 35 Western and Central PA municipalities across a total area of approximately 51,000 Km^2^ (approximately 2 million inhabitants). Since 2008, cameras have been placed in 51 offices; by 2020, 48 sites had a camera, with three sites having discontinued participation. Additional sites refer patients for DREs to sites with a camera (n = 46). This referral system has increased the total number of sites in the program to 94.

**Fig 3:**
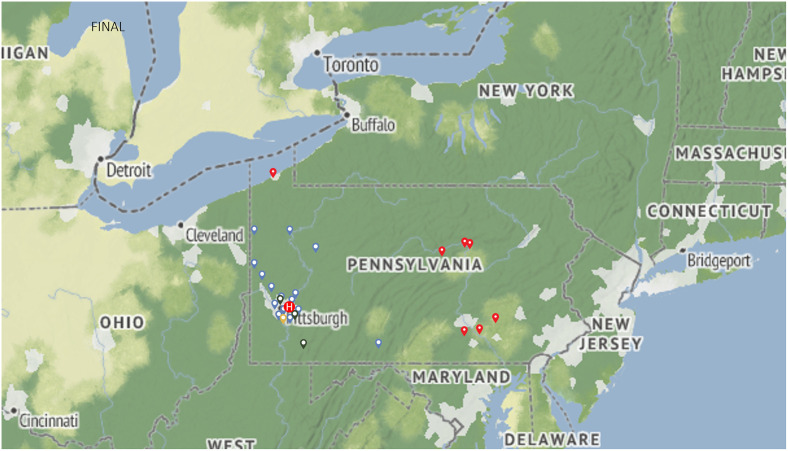
Geographic distribution of the cameras in the program. Legend: Locations marked with a blue pin are UPMC Community medicine Inc. (CMI) practices, with a black pin are University of Pittsburgh Physicians (UPP), with a red pin are remote locations (>100 miles from Pittsburgh), and with a yellow pin are non-UPMC clinics.

Reprinted from NASA Earth Observatory website under a CC BY 4.0 license, Public domain, original copyright 2024, retrieved from (https://earthobservatory.nasa.gov/map#6/41.179/-78.223).

A total of 87 sites are owned by UPMC, most of which are primary care clinics in Pittsburgh and its surrounding areas (Fig 3). One camera is stationed on a mobile unit and used to close care gaps at nursing homes and community centers; seven have been placed at sites greater than 100 miles from Pittsburgh (UPMC Annville, UPMC Susquehanna UPMC Hammot-Erie); and there are also 4 FQHC and 3 free clinics. In terms of specialties, most of the sites are focused on primary care, and 3 cameras have been placed in endocrinology clinics.

#### 2.2. Participants

From August 2008 to December 2020, there were 21,960 DREs. The monthly distribution of photographs between 2008 and 2020 is shown in Fig 1. A total of 16,458 unique individuals were examined during this period. The age average of the examined patients was 58.5 ± 13.7 years and 46.5% (7,657) were female. The majority of patients identified themselves as White (65.0%, 10,699). Only 1.5% (246) were Hispanic or Latino, almost half (44.6%, 7,342) were unmarried (divorced, legally separated, single, or widowed), and most had type 2 diabetes (93.4%). See Table 1.

**Table 1:** Characteristics of individuals in their first visit participating in diabetic retinopathy examinations (DRE) in the point of care diabetic retinopathy examination program (POCDREP) at UPMC (n = 16,458).

Characteristic	
Sex, n (%)	
Female	7,657 (46.5)
Male	8,726 (53.0)
*Missing*	73 (0.5)
Age, mean (SD)*	58.5 (13.7)
Race, n (%)	
Black	3,786 (23.0)
White	10,699 (65.0)
Other	1,973 (11.9)
Hispanic or Latino, n (%)	246 (1.5)
Non-Hispanic or Latino	14,208 (86.3)
*Missing*	2,004 (12.2)
Language, n (%)	
English	14,185 (86.2)
Spanish	105 (0.6)
Others	206 (1.2)
*Missing*	1,962 (11.9)
Marital status, n (%)	
Married (married, cohabitating)	6,820 (41.4)
Unmarried (widow, divorce)	7,342 (44.6)
*Missing*	2,296 (13.9)
Diabetes type, n (%)	
Type 1	618 (3.8)
Type 2	15,374 (93.4)
*Missing*	466 (2.8)

SD: Standard deviation.

*Age was missed in 0.8% (132) of participants.

Out of the total individuals, 75.8% (12,469) had a single DRE, 17.7% (2,909) had two, 4.6% (752) had 3, and 2% (328) had 4 or more. There was a trend of growth in the number of images until the COVID-19 pandemic, when the program was paused (Fig 1).

#### 2.3. Exams

At the time of DRE (n = 21,960, Table 2), most participants used Medicare (32.2%, 7,073) as insurance, followed by commercial insurance (29.2%, 6,406). In terms of socioeconomic status at examination based on zip codes, 6.9% (1,524) of examined patients were below the poverty line (>Q2, 20%-39.9%). None of the patients imaged lived in the zip code designated as extreme poverty areas.

**Table 2:** Characteristics of individuals when examined in the point of care diabetic retinopathy examination program (POCDREP) at UPMC (n = 21,963).

Characteristic*	
Insurance, n (%)	
Commercial	6,406 (29.2)
Medicaid	3,178 (14.5)
Medicare	7,073 (32.2)
Dual	1,572 (7.2)
Veterans’ affairs (VA)	86 (0.4)
Uninsured	227 (1.0)
*Missing*	3,421 (15.6)
Poverty quantile	
<10% (Q1)	12,632 (57.5)
10–19.9% (Q2)	5,660 (25.8)
20–29.9% (Q3)	917 (4.2)
30–39.9 (Q4)	607 (2.8)
*Missing*	2,147 (9.8)
Smoking status, n (%)	
Never smoked	8,265 (37.6)
Former smoker	6,687 (30.4)
Smoker	4,129 (18.8)
*Missing*	2,882 (13.1)
Body mass index (BMI), mean (SD)*	34.0 (8.0)
BMI categories	
Underweight (BMI<18.5)	88 (0.4)
Normal (BMI = 18.5–24.9)	1,886 (8.6)
Overweight (BMI = 25–29.9)	4,723 (21.5)
Obesity class I (BMI = 30–34.9)	5,375 (24.5)
Obesity class II (BMI = 35–39.9)	3,711 (16.9)
Obesity class III (BMI≥40)	3,917 (17.8)
*Missing*	2,263 (10.3)
HbA1c, mean (SD)*	7.6 (1.9)

SD: Standard deviation.

*BMI and HbA1c had 10.3% (2,263) and 15.2% (3,346) of missing values, respectively.

When examined, most people reported that they never had smoked (37.6%, 8,265) or were former smokers (30.4%, 6,687). Participants’ average BMI at the moment of imaging was 33.96±8.02 (n = 19,700, range 12.6–88), and their mean hemoglobin A1c (HbA1c) was 7.58±1.88 (n = 18,617, range 3.5–18.5) with 9.75% with levels ≥10% (Table 2).

*2*.*2*.*1 Findings*. From the images collected (n = 43,665), 63.3% were of good quality, 18.62 fair, 4.41% just gradable, and 2.64% of poor quality; and 10.45% were non-gradable. Only 0.6% (261) of participants’ eyes were not imaged due either to monocular status, or the staff not uploading the image for reading. Non-gradable images rates per eye were 2.7% (592) and 3.7% (828) for the right and left eye, respectively.

**Fig 4:**
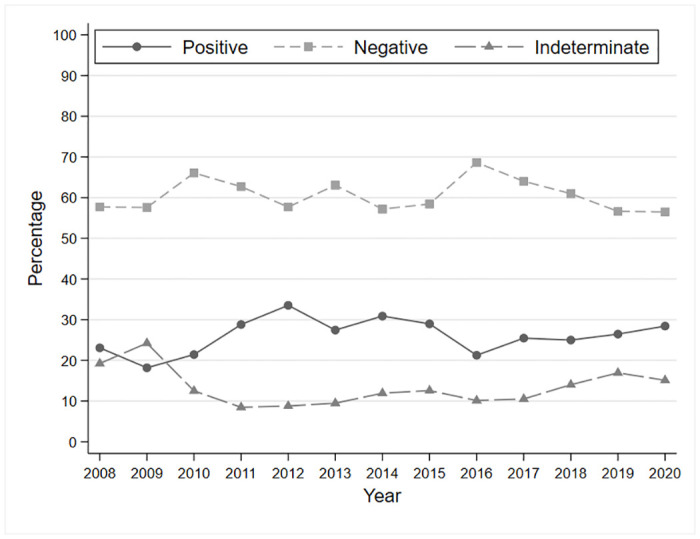
Outcomes of the diabetic retinopathy exams (n = 21,960) in terms of positive, negative and indeterminate results.

Exams were gradable in 86.3% (18,957) of the tests. The percentages of indeterminate exams (non-gradable and no image) declined in the first four years of the program and stabilized until the inclusion of many new clinics in 2017; however, the rate appears to have stabilized afterwards. Additionally, the prevalence of positive DRE findings consistently remained within the range of 20% to 30% throughout the entire program duration (Fig 4).

**Fig 5:**
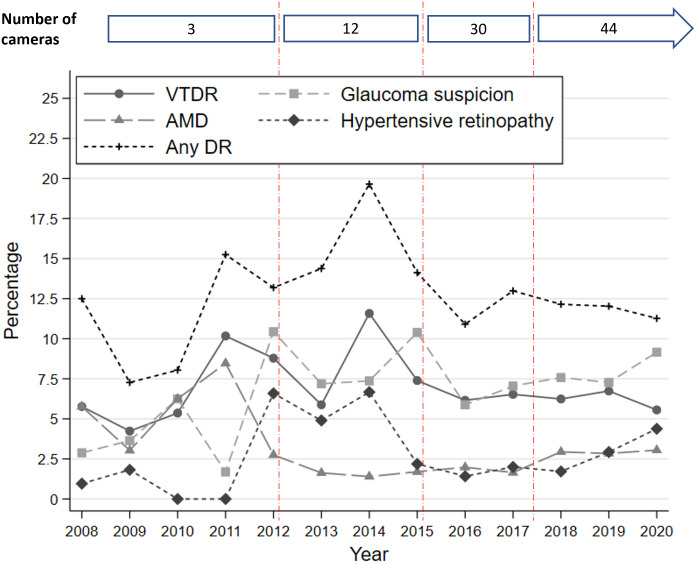
Program rate trends of diagnosis of any DR, vision-threatening diabetic retinopathy, age-related macular degeneration (AMD), glaucoma suspicion, and hypertensive retinopathy since 2008 to 2020.

Hypertensive retinopathy was diagnosed less in the first years of the program, while AMD and VTDR were the most common findings. After 2016, there appears to have been a stabilization of the percentages of these diseases with a higher incidence of VTDR and suspected glaucoma. Fig 5 shows the trends of diagnosis of VTDR, AMD, suspected glaucoma, and hypertensive retinopathy.

Almost one-third (6,928, 31.5%) of the exams resulted in a recommendation for a follow-up appointment with an ophthalmologist. This determination relied on the exam being labeled as positive, other findings necessitating follow-up, indeterminate results, or a combination of these factors. Among exams that were gradable (18,957), 59.0% were within normal limits, 12.1% showed at least some evidence of DR, 7.8% suspected glaucoma, 2.9% hypertensive retinopathy, and/or 2.7% AMD. Some images were not gradable for DR but demonstrated visible pathology other than DR, and therefore required further evaluation (2.0%, 441).

Of those exams showing a level of DR, 10.9% (2,404) had nonproliferative disease, whether mild (6.3%, n = 1,385), moderate (3.6%, n = 791), or severe (1.0%, n = 221). Proliferative disease was found in 0.8% (177) of exams, macular exudate in 3.0% (661), and panretinal photocoagulation in 0.7% (144) of exams. VTDR was identified in 6.4% (1,409) of the exams.

## Discussion

We documented the origins and evolution, current state, and outcomes of a point-of-care diabetic retinopathy examination program (POCDREP) at UPMC, an academic health center in Pittsburgh. Since 2008, the program has overcome several challenges and proven to be a user-friendly, reproducible tool for DRE with promising results across different sites and healthcare systems (i.e., FQHC and free clinics). Other programs have been previously described particularly with focus on the technology and the imaging transmission [19, 22–24, 32]; however, to the best of our knowledge, this is the first description of a program that includes not only technical aspects but emphasizes in the human factors of a DRE program, and the most extensive data review period (spanning 13 years) among programs of a similar nature.

### Program structure

One of the program’s critical features is the use of cameras that do not require a mydriatic pupil for acquiring retinal images [43, 44], facilitating image-taking in primary healthcare centers after a short course of training and supervision, followed by remote image interpretation by ophthalmologists who analyze and detect abnormalities that indicate DR.

The program has demonstrated high reproducibility as we developed a standardized workflow that ensures consistent results across different users and settings. It allows the program to be used in multiple locations, serving the entire population without regard for insurance or economic status, even in areas where access to specialist medical professionals is limited (from a low-resource point of care, free clinics to multispecialty sites).

Another highlight of this program is its participatory approach, rarely mentioned in other programs [45, 46]. Primary care sites personnel have a voice in the program to help with its continue growth and removal of barriers for examinations. They, as a team, helped with implementation and development of protocols for image transmission, provided feedback about training, imaging strategies, and established their own collaborations with other sites for them to become part of the program (i.e., loaning a camera between clinics).

### Patients’ characteristics

Based on patients’ characteristics, the program had an equal gender distribution, with a 50/50 male-to-female ratio. Their mean age is 60 years, which includes both early (<40 years) and late-onset diabetes (>40 years). Our program included a widely distributed population not limited to a specific age group, which is relevant for DR screening as the disease can affect individuals at any age and stage of their disease [47]. This real-life study also shows rates of continuity of care, where around 25% of the patients had at least another yearly exam within the program, which are similar to those previously reported [25]. Continuity of DR care is relevant as shown in a cohort study analyzing changes in DR levels using DRE over a 5-year period, where 6% of patients without retinopathy and 27% with mild non-proliferative DR progressed to VTDR [26]. Further research on the incidence of new retinopathy or its progression within the POCDREP is required.

Participants in our study reflect the Pittsburgh’s demographic composition. For instance, Medicaid recipients comprised 14.5%, closely aligning with the 16.5% reported in the 2021 census. Similarly, the representation of Veterans Affairs beneficiaries at 0.4% mirrors the census figure of 0.91%. Despite challenges with sustainability and implementation, we reached a part of the uninsured segment of the population (1%); however, not enough when the 2021 Census described a 5.46% rate of uninsured people [48]. Barriers to obtaining specialty care for uninsured patients encompassed out-of-pocket costs, stigma, scarcity of local specialists accepting uninsured patients, and logistical challenges like transportation. Consequently, teleophthalmology in primary care emerges as a tool to address these barriers and bridge healthcare gaps not only for uninsured patients but also for those with limited coverage [49].

In the US, Hispanic and Black patients have higher prevalence of DR and VTDR.[50] As the patients within this study mostly identified as White, further research and quality improvement efforts are needed to reach high-risk populations.

Obesity, is a well-recognized, prevalent risk factor for multiple diseases, including type 2 diabetes, and can be a predictor and monitoring parameter for diabetic patients [51]. Patients participating in the POCDREP were found to have an average BMI of 34±8 and only 8.6% of the population had a normal BMI. A 2021 US study, demonstrates a direct correlation between obesity and increased prevalence of severe DR, including VTDR [52]. Furthermore, obesity has a significant correlation with multiple microvascular (retinopathy, neuropathy, nephropathy) complications in diabetic patients, thereby emphasizing the need for sustainable strategies to prevent obesity and its complications [53].

The HbA1c goal in diabetic patients varies depending on individual factors such as age, comorbidities, and diabetes duration [54]. However, in general, the American Diabetes Association recommends an HbA1c target of less than 7% for most non-pregnant adults with diabetes [55]. Diabetic patients with an HbA1c ≥ 7% are 6.9 times more likely to develop DR than those with an HbA1c < 7%, including VTDR [56]. During the program, we observed individuals had an average hemoglobin A1c of 7.6 ±1.9, with almost 10% of patients with levels above 9.9%, which suggests inadequate disease control and an increased risk of microvascular complications such as VTDR [54].

### Program outcomes

Non-gradable exams in our study (10.5% either or both eyes) were similar to those reported in similar DR programs in the US and internationally [8, 17, 20, 23, 25, 27–31]. The use of dilating drops has been suggested as a way to overcome this barrier, a strategy that has been implemented with training recently in our program. However, certain diabetic patients encounter multiple constraining factors for acquiring good quality retinal images even after pupil dilation, such as cataracts, smoking, and small pupils due to an autonomic neuropathy [17, 57, 58].

Approximately one-third of individuals examined for DR demonstrated positive results, indicating a substantial prevalence of the condition in asymptomatic individuals. VTDR, AMD, glaucoma suspicion [59], were identified as the most common findings which is similar to global literature on the topic [8, 17, 20, 23, 25, 27–31]. Implementing secondary prevention programs may improve prognosis and disease control while also helping identify other prevalent causes of blindness [60]. This may also reduce the incidence of vision loss in diabetic patients, thereby reducing the cost of treatment by preventing complications.

A follow-up appointment with an ophthalmologist was requested in 31.5% of the exams. In the U.S., research indicates that less than 40% of patients with diabetes receive annual eye examinations, and this rate is even lower among underserved populations [61, 62]. Even in the population already receiving treatment for proliferative DR, the non-follow-up rate is 16.3% [63, 64]. Further research is needed to identify follow-up rates and the factors preventing patients from adhering to the recommendations after a positive exam.

As for the revenue generated by this program, conducting a cost analysis to evaluate the impact of early disease detection on overall costs could provide valuable insights for healthcare providers and policymakers. Researchers can better understand the cost-effectiveness of such interventions by assessing the costs associated with early disease detection and comparing them to the potential savings from preventing or reducing the severity of DR [65]. Identifying the impact of DRE using non-mydriatic retinal cameras in Pennsylvania also has the potential to support the expansion of such programs and serve as an impetus for further research in this domain. These initiatives will necessitate collaboration between healthcare providers, policymakers, and researchers to develop comprehensive approaches to prevent DR.

### Limitations

Retrospective studies have limitations that must be considered when interpreting their results. One of the primary limitations of our study was the lack of an early standardization of the program data collection tools, which limited the number of variables to analyze in the full cohort of patients, including information on their follow-up status. The implications of information loss are twofold: it can substantially influence the statistical robustness of the study and restrict the applicability of its outcomes to a broader context. Nonetheless, we rigorously sought to mitigate this limitation. Thorough scrutiny of available data sources was conducted to maximize the information gleaned from the program. This meticulous approach aimed to minimize potential biases that could result from the loss of information, thereby enhancing the reliability and validity of our study’s findings. The absence of data on follow-up assessments during the first decade of the program prevented us to compare rates between different implementation periods. Further research is being carry out to identify factors associated with lost to follow-up after the exams. The issue of missing data can be fixed with a prospective data collection method or a registry that includes more variables and continuously captures accurate data about the patient and the program.

## Conclusions

The Point of Care Diabetic Retinopathy Examination Program (POCDREP) has successfully reached comparable outcomes with other national and international DRE programs. The POCDREP has proven to be a reproducible tool for detecting DR with promising results within a range of point-of-care sites. Sustainability of the program can be attributed to the development of explicit guidelines, building a participatory and collaborative network of stakeholders from participating primary care sites, ongoing training for healthcare providers in performing DRE, and the establishment of monitoring systems to track DREs over time. Considering that the program primarily focuses on DRE, the detection of other diseases enhances overall healthcare.

## Supporting information

S1 File IRB approval STUDY20010159. (PDF)

S2 File Quality improvement project. (PDF)

S3 File Teleophthalmology studies analysis. (XLSX)
