# Supplementary material for: Comprehensive insights into a decade-long journey: The evolution, impact, and human factors of an asynchronous telemedicine program for diabetic retinopathy screening in Pennsylvania, United States
Source: PLoS One. 2024 Jul 12;19(7):e0305586. doi: 10.1371/journal.pone.0305586 (PMC11244789; doi:10.1371/journal.pone.0305586)
Supplement: S1 File — (PDF) [file pone.0305586.s001.pdf]

University of Pittsburgh
Institutional Review Board

Human Research Protection Office
3500 Fifth Avenue, Suite 106
Pittsburgh, PA 15213
Tel (412) 383-1480
www.hrpo.pitt.edu

APPROVAL OF SUBMISSION (Exempt)

Date:	May 22, 2020
IRB:	STUDY20010159
PI:	Evan Waxman
Title:	Development of a telemedicine program to prevent diabetic retinopathy in Pennsylvania
Funding:	None

The Institutional Review Board reviewed and approved the above referenced study. The study may begin as outlined in the University of Pittsburgh approved application and documents.

Approval Documentation

Review type:	Initial Study
Approval Date:	5/22/2020
Exempt Category:	(4) Secondary research on data or specimens (no consent required)

Determinations:	• Waiver of HIPAA authorization
Approved Documents:	• Data collection form_development.xlsx<br>• 2020 04 10 - HRP-723 - WORKSHEET - Exemption_Secondary Data.Specimens_Version_Version_0.02.docx<br>• References

As the Principal Investigator, you are responsible for the conduct of the research and to ensure accurate documentation, protocol compliance, reporting of possibly study-related adverse events and unanticipated problems involving risk to participants or others. The HRPO Reportable Events policy, Chapter 17, is available at http://www.hrpo.pitt.edu/.

Clinical research being conducted in an UPMC facility cannot begin until fiscal approval is received from the UPMC Office of Sponsored Programs and Research Support (OSPARS).

If you have any questions, please contact the University of Pittsburgh IRB Coordinator, Emily Bird.

Please take a moment to complete our Satisfaction Survey as we appreciate your feedback.
