# Supplementary material for: Comprehensive insights into a decade-long journey: The evolution, impact, and human factors of an asynchronous telemedicine program for diabetic retinopathy screening in Pennsylvania, United States
Source: PLoS One. 2024 Jul 12;19(7):e0305586. doi: 10.1371/journal.pone.0305586 (PMC11244789; doi:10.1371/journal.pone.0305586)
Supplement: S2 File — (PDF) [file pone.0305586.s002.pdf]

QI Project Submission

Submit Cancel

Check your UPMC email for a message containing a link to your saved/submitted project.

Project Description

5

Expand section to see details

6

Project ID: 630

Project Title:

Sponsor: Waxman, Evan;

Enter your UPMC network login OR your last name, first name OR use the search icon to find your name.

Sponsor Title: Faculty - Clinician, Physician

Copy and paste from global email, e.g. "Faculty - Clinician, Physician, UPP17 Pathology Presby"

Sponsor Department: Ophthalmology

Co-Sponsor(s):

Enter your UPMC network login OR your last name, first name OR use the search icon to find your name.

Non-UPMC Co-Sponsors (enter name (email) for any non-UPMC co-sponsors):

QI Topic

Please specify the main area associated with your QI project from the list below:
▼

GME Involvement: **Are any of the sponsors or co-sponsors residents or fellows?** No ▼

Nursing Involvement **Are any of the sponsors or co-sponsors nurses?** No ▼

ICU **Is this an ICU-related project?**
▼

Academic Requirement: **Does this project fulfill an academic requirement?**
▼

Facility Where Project Will be Implemented:

- [ ] The Beckwith Institute
- [ ] Children's Hospital of Pittsburgh of UPMC
- [ ] ISMETT
- [ ] Magee-Womens Hospital of UPMC
- [ ] Patient Blood Management
- [ ] Physician Services
- [ ] UPMC Altoona
- [ ] UPMC Bedford Memorial
- [ ] UPMC Cancer Centers
- [ ] UPMC Centers for Rehab Services
- [ ] UPMC East
- [ ] UPMC Hamot
- [ ] UPMC Health Plan
- [ ] UPMC Horizon
- [ ] UPMC McKeesport
- [ ] UPMC Mercy
- [ ] UPMC Northwest
- [ ] UPMC Passavant
- [x] UPMC Presbyterian
- [ ] UPMC Senior Communities
- [x] UPMC Shadyside
- [ ] UPMC St. Margaret
- [ ] Western Psychiatric Institute and Clinic
- [ ] Wolff Center
- [x] Other

Other Facility Name: Approximately 15 outpatient [illegible]

**Anticipated Start Date**	7/1/2016	**Anticipated End Date**	6/30/2018

Referred by IRB? No

Project Summary **Briefly summarize the background of the problem and aims of the project:**

The Guerrilla Eye Service Reconnaissance program (GES Recon) has deployed retina cameras to 15 primary care sites to allow early detection of sight threatening diabetic retinopathy. Over the last two years, GES Recon has been successful in detecting patients at risk vision loss and blindness. A minority of the patients identified at risk though are documented to follow through for further evaluation and treatment.

Primary aim: Improve follow through rate for patients with abnormal screening photos. We will improve the follow through rate to 80%.

Secondary aims:
1) Increase use of cameras currently deployed.
2) Identify patient barriers to follow through.

Describe the sample and setting in which the project will be conducted:

As described above

Describe the intervention and how it will be implemented:

We will employ a coordinator for the Guerrilla Eye Service Recon program to improve follow through rates, increase the use of the cameras currently deployed and identify barriers to patient follow through. A strong candidate for the coordinator position has been identified.

Identify process and outcome measures that will be used to evaluate the effectiveness of the intervention and how consistently it is implemented:

Primary aim: Follow through rate as measured as percentage of patients identified with abnormal screening photos that have documented follow through evaluations and/or treatment.

Secondary aims:
1) Number of patients screened by the GES Recon program

2) A list of the patient barriers identified, the frequency with which they are cited by patients and the importance of these as rated by patients.

Specify data collection methods -- what data will be collected, how, and by whom:

Results of camera findings. Statistics for use of cameras at each site. Follow through data.

Outline plans to maintain data security and patient confidentiality:

All data will be kept on UPMC M-Drive.

Describe methods for data analysis:

Currently being worked out.

Keywords

Please list 3-4 key search terms/phrases (e.g., falls, hand hygiene, ICU family communication, noise)

Diabetic retinopathy, Blindnes

Project Results

Enter results after project is complete.

Project Methodology

5

Expand section to see details

6

1. Corrective Plan

Is there a commitment to implementing a plan for follow-up based on the outcomes of the project?

Yes ▼ *

Description

Our plan is to demonstrate that the project saves money in the long run and to have the coordinator position funded after the study period.

2. Funding

Will this project be supported by funding or any in-kind donations such as equipment loan?

No ▼

3. Product, Device, Medication, Biologic, or Technology Evaluation:

Does the project involve the evaluation of a product, device, medication, biologic, or technology? (Check all that apply)

No ▼

4. External Participants

Are any entities external to UPMC participating in the project?

Yes ▼

List the entities and specify their role in the project:

Several of the primary care sites with cameras are not within the UPMC network.

**5. Research Association**	**Is this project associated with a University of Pittsburgh or UPMC research project or clinical trial or being conducted in order to collect pilot data that will be used as a basis for future funding proposals to external agencies or companies?**<br><br>No ▼
**6. Patient Data Collection Methodology**	**Will Patient Data be collected as part of this project?**<br>Yes ▼

Please indicate how patient data will be collected:

- [ ] **Chart Review through hard-copy medical records**
- [x] **Chart review through electronic medical records**

Please specify EMR: EPIC ▼

- [ ] **Data collection from a UPMC registry or other database**
- [ ] **Data collection from other applications**
- [ ] Patient interviews/observations

Will you be collecting protected health information (PHI) as part of this project? Yes ▼

All patient identifiable data collected and stored for this study needs to comply with UPMC Policy HS-MR1000 Release of Protected Health Information regarding the privacy and security of clinical data.

Is participation in this project/program part of a national registry or database?

No ▼

QI projects and/or programs that include the submission of local data to external partners and third parties (e.g. national registries & databases) require business associate agreements (BAA) and data usage agreements (DUA).

Please specify who will be accessing this data, including any individuals not employed by UPMC:

Noone not employed by UPMC will be accessing the data.

Please attach a sample data collection form: Click here to attach a file

data collection form.txt

7. Blinding	Does the project involve therapeutic intervention?<br>No ▼
8. Withdrawing Treatment	Does the project involve “withdrawing” or holding back any needed and generally accepted treatments for the patients’ condition?<br>No ▼
9. Randomization	Does the project involve prospective assignment of patients to different procedures or therapies based on predetermined plans such as randomization?<br>No ▼
10. Conflict of Interest	Are there any potentially relevant financial and non-financial conflicts of interest of individuals leading this project?

For illustrative purposes, potential conflicts of interest could arise from the following types of relationships, among others.

- consulting agreement between individual leading the proposed project and the manufacturer of the device or drug used or evaluated in connection with the project;
- participation by an individual involved in the proposed project on a scientific advisory board of the manufacturer of the device or drug used or evaluated in connection with the project;

- **royalty arrangement between an individual involved in the proposed project on a scientific advisory board of the manufacturer of the device or drug used or evaluated in connection with the project;**
- **Individual leading the proposed project developed intellectual property being used or evaluated as part of the project, regardless of whether such intellectual property was sold, transferred or licensed to a third party.**

No ▾

**11. QI versus Research Description**	**Briefly describe why you think this is a QI project and not a research study:**<br>We're working to measure and improve the efficacy of an existing project.
**Attach related files**	**Attach any files that support your project documentation:**<br>Click here to attach a file<br>data collection form.txt
**QRC Approval Information**	**QRC Approval Date:** 7/5/2016<br>**QRC Approval Status:** Approved

***Results of quality improvement projects must be reviewed by local quality directors and approved by the Chief Quality Officer prior to dissemination (via presentation or publication) outside of UPMC. The UPMC QRC has adopted reporting guidelines for QI studies. If the sponsor desires to publish project findings, the SQUIRE guidelines will serve as the required reporting format.*
Projects reviewed and approved by the UPMC Quality Improvement Review Committee do not meet the federal definition of research according to 45 CFR 46.102(d) and do not require additional IRB oversight.

Submit Cancel

You may need to click the "Submit" or "Save" button more than once. When your project has been successfully saved or submitted, this form will automatically close. Check your UPMC email for a message containing a link to your saved/submitted project.
